# Heterologous Production and Characterization of Two Glyoxal Oxidases from Pycnoporus cinnabarinus

**DOI:** 10.1128/AEM.00304-16

**Published:** 2016-07-29

**Authors:** Marianne Daou, François Piumi, Daniel Cullen, Eric Record, Craig B. Faulds

**Affiliations:** a Aix Marseille Université, INRA, BBF (Biodiversité et Biotechnologie Fongiques), Marseille, France; b USDA, Forest Products Laboratory, Madison, Wisconsin, USA; HKI and University of Jena

## Abstract

The genome of the white rot fungus Pycnoporus cinnabarinus includes a large number of genes encoding enzymes implicated in lignin degradation. Among these, three genes are predicted to encode glyoxal oxidase, an enzyme previously isolated from Phanerochaete chrysosporium. The glyoxal oxidase of P. chrysosporium is physiologically coupled to lignin-oxidizing peroxidases via generation of extracellular H_2_O_2_ and utilizes an array of aldehydes and α-hydroxycarbonyls as the substrates. Two of the predicted glyoxal oxidases of P. cinnabarinus, GLOX1 (*Pci*GLOX1) and GLOX2 (*Pci*GLOX2), were heterologously produced in Aspergillus niger strain D15#26 (*pyrG* negative) and purified using immobilized metal ion affinity chromatography, yielding 59 and 5 mg of protein for *Pci*GLOX1 and *Pci*GLOX2, respectively. Both proteins were approximately 60 kDa in size and N-glycosylated. The optimum temperature for the activity of these enzymes was 50°C, and the optimum pH was 6. The enzymes retained most of their activity after incubation at 50°C for 4 h. The highest relative activity and the highest catalytic efficiency of both enzymes occurred with glyoxylic acid as the substrate. The two P. cinnabarinus enzymes generally exhibited similar substrate preferences, but *Pci*GLOX2 showed a broader substrate specificity and was significantly more active on 3-phenylpropionaldehyde.

**IMPORTANCE** This study addresses the poorly understood role of how fungal peroxidases obtain an *in situ* supply of hydrogen peroxide to enable them to oxidize a variety of organic and inorganic compounds. This cooperative activity is intrinsic in the living organism to control the amount of toxic H_2_O_2_ in its environment, thus providing a feed-on-demand scenario, and can be used biotechnologically to supply a cheap source of peroxide for the peroxidase reaction. The secretion of multiple glyoxal oxidases by filamentous fungi as part of a lignocellulolytic mechanism suggests a controlled system, especially as these enzymes utilize fungal metabolites as the substrates. Two glyoxal oxidases have been isolated and characterized to date, and the differentiation of the substrate specificity of the two enzymes produced by Pycnoporus cinnabarinus illustrates the alternative mechanisms existing in a single fungus, together with the utilization of these enzymes to prepare platform chemicals for industry.

## INTRODUCTION

Lignocellulose degradation is a key step in carbon recycling. Due to the importance of lignocellulosic material as a renewable source of platform chemicals and a potential substitute for fossil-derived ones, there is increasing interest in improving the efficiency of the use of lignocellulose through industrial biotechnology approaches. Various naturally occurring microorganisms are well adapted to degrade the cellulose, hemicellulose, and lignin components of the lignocellulosic biomass and use them as carbon and energy sources (1). White rot fungi are particularly adapted to degrade lignin, the most recalcitrant polymer in biomass, through the production of a group of extracellular lignin-degrading enzymes, such as class II peroxidases, laccases, and peroxide-generating enzymes, such as aryl alcohol oxidases and glyoxal oxidases (2).

Phanerochaete chrysosporium was for years the most studied white rot lignin-degrading fungus, and these studies contributed to most of the current understanding of the enzymology behind lignin degradation. However, the recent expansion in genome analyses has shown that the repertoire of genes varies substantially among white rot fungi. For example, P. chrysosporium lacks genes encoding laccases but features 15 class II peroxidases and a single glyoxal oxidase gene (3), whereas a recent analysis has identified 5 laccase, 9 class II peroxidase, and 3 glyoxal oxidase genes in Pycnoporus cinnabarinus (4). Extracellular H_2_O_2_-generating enzymes, such as glyoxal oxidase (GLOX), are thought to be involved in lignin degradation by providing hydrogen peroxide, which is necessary for peroxidase activity (5). Other fungal enzymes demonstrated to generate H_2_O_2_ include sugar oxidases, methanol oxidase, and aryl-alcohol oxidase (6). These auxiliary activity (AA) enzymes (7) have been classified within the Carbohydrate-Active Enzymes database (CAZy; http://www.cazy.org), where they are currently divided into 13 families on the basis of their catalytic modules, reaction mechanisms, and substrate specificities.

In this classification, copper radical oxidases (CROs) form the family AA5, all of which are characterized by the presence of a free radical-coupled copper ion acting as a two-electron redox catalytic site (8). Subfamily AA5_1 includes GLOX and structurally related copper CROs, whereas subfamily AA5_2 contains galactose oxidases (7). Although GLOX and galactose oxidase share a conserved active site, the enzymes differ in their number of domains, redox potential, free radical stability, and substrate specificity (5). Beyond GLOX, other members of the AA5_1 subfamily (CRO) differ from GLOX by their structure, regulation, and, at least in one case, substrate specificity (9).

GLOX is responsible for the oxidation of aldehydes, leading to the formation of carboxylic acids and the simultaneous reduction of dioxygen to hydrogen peroxide (8):

$$\text{RCHO}+{\text{O}}_{2}+{\text{H}}_{2}\text{O}\to {\text{RCO}}_{2}\text{H}+{\text{H}}_{2}{\text{O}}_{2}$$

They catalyze the oxidation of aldehydes and alpha-hydroxycarbonyls derived from carbohydrates (10) and lignin degradation (5). Some of these substrates have been found in cultures of lignin-degrading fungi; therefore, they are likely to be the *in vivo* substrates for GLOX (9). GLOX also can act as a detoxifying agent during biomass breakdown and generate products favoring enzymatic degradation (8, 11). However, only the GLOX proteins from P. chrysosporium and Ustilago maydis have been characterized to date (12, 13), and more studies are needed to understand the role of these enzymes.

Seven genes encoding enzymes belonging to the AA5_1 family, including 3 GLOX enzymes and 4 CROs, were previously identified in the genome of P. cinnabarinus (4). However, only two of the GLOX enzymes (P. cinnabarinus GLOX1 [*Pci*GLOX1] and P. cinnabarinus GLOX2 [*Pci*GLOX2]) were apparently secreted into the culture medium under the defined growth conditions, as identified through proteomic analysis of the secretome (4). This study describes the production and characterization of these enzymes.

## MATERIALS AND METHODS

### Strains and culture conditions.

P. cinnabarinus strain BRFM137 was obtained from the Centre International de Ressources Microbiennes-Champignons Filamenteux (CIRM-CF; https://www6.inra.fr/cirm_eng/Filamentous-Fungi). Aspergillus niger strain D15#26 (*pyrG* negative) (14) was used for the heterologous expression of the synthesized coding genes of the two *Pci*GLOX enzymes. The *pyrG* gene encodes orotidine-5′-phosphate decarboxylase, which is an enzyme essential for the biosynthesis of pyrimidine nucleotides, and thus, mutants lacking this enzyme require uracil or uridine for growth (15). Escherichia coli strain JM109 (Promega, Charbonnieres, France) was used for vector storage and propagation and was cultured on 2YT medium (85 mM NaCl, 1% yeast extract, 1.6% Bacto tryptone) containing ampicillin (final concentration, 100 μg/ml).

A. niger transformants were isolated on the basis of selective growth on uridine-free solid minimal medium, which contained MgSO_4_·7H_2_O (2 mM), sorbitol (0.8 M), glucose (55.5 mM), NaNO_3_ (70 mM), KCl (7 mM), KH_2_PO_4_ (11 mM), and 1× trace elements solution (the 1,000× stock solution contained ZnSO_4_·7H_2_O [76 mM], H_3_BO_3_ [178 mM], MnCl_2_·4H_2_O [25 mM], FeSO_4_·7H_2_O [18 mM], CoCl_2_·6H_2_O [7.1 mM], CuSO_4_·5H_2_O [6.4 mM], Na_2_MoO_4_·2H_2_O [6.2 mM], and EDTA-Na_2_·7H_2_O [174 mM]). Positive transformants were cultured in liquid production medium containing NaNO_3_ (70 mM), KCl (7 mM), glucose (277.5 mM), MgSO_4_·7H_2_O (2 mM), 1× trace elements solution, Na_2_HPO_4_ (200 mM), and vitamin solution. The pH of the production medium was adjusted to 5.5 with 1 M citric acid prior to inoculation and maintained at this pH during growth.

### Cloning and transformation.

The genes coding for *Pci*GLOX1 and *Pci*GLOX2 (scf184747.g48 and scf184747.g42, respectively) were previously identified in the sequenced genome of P. cinnabarinus (4). The open reading frames (ORFs) of the two genes were predicted from their genomic sequences by translating the six frames and identifying splicing sites according to the general GTRMGT … YAG pattern. This prediction was further supported using the Augustus gene prediction website (http://bioinf.uni-greifswald.de/augustus/) and by aligning the sequence of the ORF with the sequences of similar genes from other organisms using tblastx (NCBI). The signal peptides of *Pci*GLOX1 (MKWTSLSLLPLLAPLALG) and *Pci*GLOX2 (MFQTTLHLLFVLVVTGRGLA) were replaced by the 24-amino-acid glucoamylase (GLA) prepro sequence from A. niger (MGFRSLLALSGLVCNGLANVISKR). A sequence encoding a 6×His tag was also added downstream of the coding region of the genes to facilitate protein purification. The cDNA sequences of the two *Pci*GLOX enzymes containing the above-mentioned modifications were codon optimized for A. niger expression and synthesized (GeneArt; Thermo Fisher Scientific, Saint Aubin, France). The synthetic genes were then cloned in the HindIII/MluI site of the pAN52.4 vector. The vectors were propagated in E. coli strain JM109 (Promega). A. niger was cotransformed following the protocol described by Punt and van den Hondel (16) with pAB4.1 containing the *pyrG* gene and pAN52.4 vectors containing the recombinant genes. Positive transformants were isolated on minimal medium on the basis of their ability to grow in the absence of uridine.

### Protein production.

Thirty different clones of each of *Pci*GLOX1 and *Pci*GLOX2 along with two negative controls were cultured to check protein production by enzymatic activity tests. Nine clones of *Pci*GLOX1 and 13 clones of *Pci*GLOX2 tested positive for GLOX activity. No activity was detected in the negative controls. For each of the two proteins, two positive clones showing maximum activity were selected, and the corresponding proteins were further purified using His SpinTrap columns (GE Healthcare Life Sciences, Buc, France). The protein produced was analyzed by SDS-PAGE and Western blotting in order to verify the presence of the 6×His tag. From the positive clones, one clone each of *Pci*GLOX1 and *Pci*GLOX2 was used for large-scale production. About 4 × 10^6^ spores from the positive clones, stored in physiological water, were used to inoculate 100 ml of production medium in 250-ml baffled flasks. Eight flasks were prepared for each protein. The cultures were then incubated at 30°C with shaking at 130 rpm for 11 days. The pH of the cultures was monitored every day and adjusted when needed to pH 5.5 using sterilized citric acid (1 M).

### Enzyme purification.

The culture media were harvested on day 11 of growth, pooled, and filtered through Miracloth (Merck Millipore, Darmstadt, Germany). The filtrate (600 ml) was collected and filtered through glass fiber filter (GF/D, GF/A, and GF/F; Whatman; GE Healthcare Life Sciences) and a 0.22-μm-pore-size polyethersulfone membrane (Express Plus; Merck Millipore). Filtrates were concentrated to 100 ml using a Vivacell 250 concentration unit with a 10-kDa-molecular-mass-cutoff polyethersulfone membrane (Sartorius, Aubagne, France). The pH of the concentrated filtrate was adjusted to 7.8 with NaOH (1 M) and sterile filtered (pore size; 0.22 μm; Express Plus; Membrane Millipore) again. His-tagged proteins were purified by immobilized metal affinity chromatography (IMAC) using an Äkta purifier (GE Healthcare Life Sciences). The samples were loaded onto a 5-ml HisTrap HP column prepacked with Ni Sepharose and equilibrated with binding buffer (50 mM Tris-HCl, pH 7.8, 150 mM NaCl, 10 mM imidazole). The His-tagged proteins were gradually eluted with 30% and 100% elution buffer (50 mM Tris-HCl, pH 7.8, 150 mM NaCl, 500 mM imidazole). Recovered recombinant proteins were then dialyzed against 50 mM sodium phosphate, pH 7. The total protein concentration was determined by the Bradford assay using a Bio-Rad protein assay kit (Bio-Rad, Marnes-la-Coquette, France) and bovine serum albumin as a standard, and the concentration of the purified proteins was determined spectrophotometrically at 280 nm using a NanoDrop 2000 spectrophotometer (Thermo Fisher Scientific).

### Protein characterization.

Purified proteins were fractionated on a 12% SDS-polyacrylamide gel, which was then stained with Coomassie blue. The molecular mass of the proteins was estimated according to the standard markers PageRuler prestained protein ladder (10 to 180 kDa; Thermo Fisher Scientific).

For the Western blot analysis, proteins were blotted onto a nitrocellulose membrane (Invitrogen, Cergy-Pontoise, France). The membrane was blocked at 4°C overnight in Tris-NaCl, pH 7.4, containing 5% semiskimmed milk powder. A 1:2,000 dilution of monoclonal antipolyhistidine antibody (Sigma-Aldrich, Saint-Quentin Fallavier, France) was then added, and the membrane was incubated for 1.5 h at room temperature on a benchtop shaker. The membrane was then washed 3 times with Tris-NaCl, pH 7.4, containing 0.05% Tween 20. Bound antibodies were visualized by staining with nitroblue tetrazolium and 5-bromo-4-chloro-3′-indolylphosphate (Roche, Meylan, France).

The isoelectric point of both proteins was predicted by use of the ProtParam-ExPaSy tool (accessible at http://web.expasy.org/protparam) and was confirmed by use of an isoelectric focusing (IEF) gel per the manufacturer's protocol (Bio-Rad). Fifteen micrograms of protein was migrated on a pH 3 to 10 Criterion IEF precast gel (Bio-Rad). IEF standards with a pI range of 4.45 to 9.6 (Bio-Rad) were used as markers.

Deglycosylation of pure *Pci*GLOX1 and *Pci*GLOX2 was performed using peptide-*N*-glycosidase F (PNGase F; New England BioLabs, Evry, France) following the manufacturer's denaturing protocol. Eight micrograms of each protein was deglycosylated, and the extent of deglycosylation was analyzed by SDS-PAGE, as described above. The N-terminal sequence was determined by Edman degradation. Analysis was carried out on an Applied Biosystems 470A sequencer, where phenylthiohydantoin amino acids were separated by reverse-phase high-performance liquid chromatography (HPLC).

### Enzyme activity.

The H_2_O_2_-dependent oxidation of 2,2′-azinobis(3-ethylbenz-thiazoline-6-sulfonic acid) (ABTS; Sigma-Aldrich) by horseradish peroxidase (HRP; Sigma-Aldrich) was used to measure GLOX activity, as previously described (12, 17) with modifications. Methylglyoxal (Sigma-Aldrich) was used as the substrate, unless otherwise stated. The reaction mixture contained 50 mM sodium 2,2-dimethylsuccinate, pH 6, 8 units of type II HRP, 0.1 mM ABTS, 10 mM methylglyoxal, and purified *Pci*GLOX enzymes in a final volume of 1 ml. When activity was measured, a small amount of H_2_O_2_ (5 μM) was also added to eliminate the lag period, as described previously (12). The reaction was initiated by the addition of methylglyoxal, and the activity was followed at 30°C for 1.5 min by measuring the absorbance at 420 nm. Measurements in all experiments were performed in triplicate, and means and standard deviations were determined. Substrate specificity was determined by the same assay but with the methylglyoxal being replaced by 10 mM the substrates listed in Table 2. The effect of copper on enzyme activity was determined by measuring enzyme activity before and after preincubation of the enzyme with 1 mM CuSO_4_ for 30 min at 800 rpm and 25°C (17).

### Temperature and pH effects.

The optimum temperature against methylglyoxal was determined from 25°C to 80°C in 5°C increments. The pH optimum was measured in sodium 2,2-dimethylsuccinate buffer over a pH range of 4.0 to 6.0 in increments of 0.5 pH units. The use of HRP as the second enzyme in this coupled reaction did not alter the results since the pH range of HRP activity is 4.0 to 8.0 (18). The thermal stability of both enzymes was determined by incubating the proteins at 30, 40, 50, 60, 70, and 80°C. The enzyme was cooled in ice before the activity was measured. Similarly, the pH stability was analyzed by incubating the enzyme in 2,2-dimethylsuccinate buffer at pH 4, 4.5, 5, 5.5, and 6. For both experiments, the activity was measured after 15, 30, 60, 120, and 240 min of incubation.

### Steady-state kinetics.

The kinetic constants for the two *Pci*GLOX enzymes were measured following the standard activity test described above by using methylglyoxal (0.3 to 10 mM for *Pci*GLOX1 and 0.01 to 10 mM for *Pci*GLOX2), glyoxal (0.3 to 10 mM), glyoxylic acid (0.005 to 10 mM), and glycerol (20 to 550 mM for *Pci*GLOX1 and 150 mM for *Pci*GLOX2) as the substrates. Lineweaver-Burk plots, obtained using the GraFit (version 4) program, were used to calculate the kinetic parameters (19).

### HPLC analysis.

The products of the oxidation of glyoxylic acid, methylglyoxal, and glyoxal by GLOX were analyzed by HPLC. Reaction mixtures were separated on an Aminex HPX-87H column (300 by 7.8 mm; Bio-Rad) at 45°C with 2.5 mM sulfuric acid as the mobile phase at a flow rate of 0.5 ml/min. Eluted compounds were detected using a refractive index detector. The reaction mixtures, which contained 10 mM oxidase substrate, were prepared as described above and incubated overnight at 30°C. Controls contained the same components but not GLOX. All reactions were filtered through 10-kDa-molecular-mass-cutoff Nanosep polyethersulfone membrane columns (Pall Corporation, Saint-Germain-en-Laye, France) and 0.45-μm-pore-size polyvinylidene difluoride syringe filters (Restek, Lisses, France) before injection in the column.

### Phylogenetic study and sequence alignments.

Copper radical oxidase protein models were downloaded from the Joint Genome Institute (JGI) web portals for the Polyporales Trametes versicolor, Dichomitus squalens, Ganoderma sp., Phanerochaete carnosa, Bjerkandera adusta, and Phlebia brevispora. CRO sequences for the Polyporales Phanerochaete chrysosporium were derived from GenBank (9), and the Pycnoporus cinnabarinus sequences are described in this work. Also retrieved from the JGI were models for a representative Agaricales, Pleurotus ostreatus; Agaricus bisporus, a member of the Auriculariales; Auricularia delicata; the Corticales species Punctularia strigozonata; and a Russulales, Stereum hirsutum. All sequences were manually trimmed of secretion signals and, where present, N-terminal repeats of the WSC domain (a cell wall stress-response component). Alignments and phylogenetic trees were constructed using the ClustalW program within the MegAlign module (version 11.0.0) of DNAStar software (Madison, WI). Bootstrap values were generated with 1,000 trials, and the Kimura distance formula was used to calculate amino acid substitutions.

### Accession number(s).

The sequences of *Pci*GLOX1 and *Pci*GLOX2 are available in GenBank under accession numbers KU215437 and KU215438, respectively.

## RESULTS

### Phylogenetic analysis and protein production.

**FIG 1:**
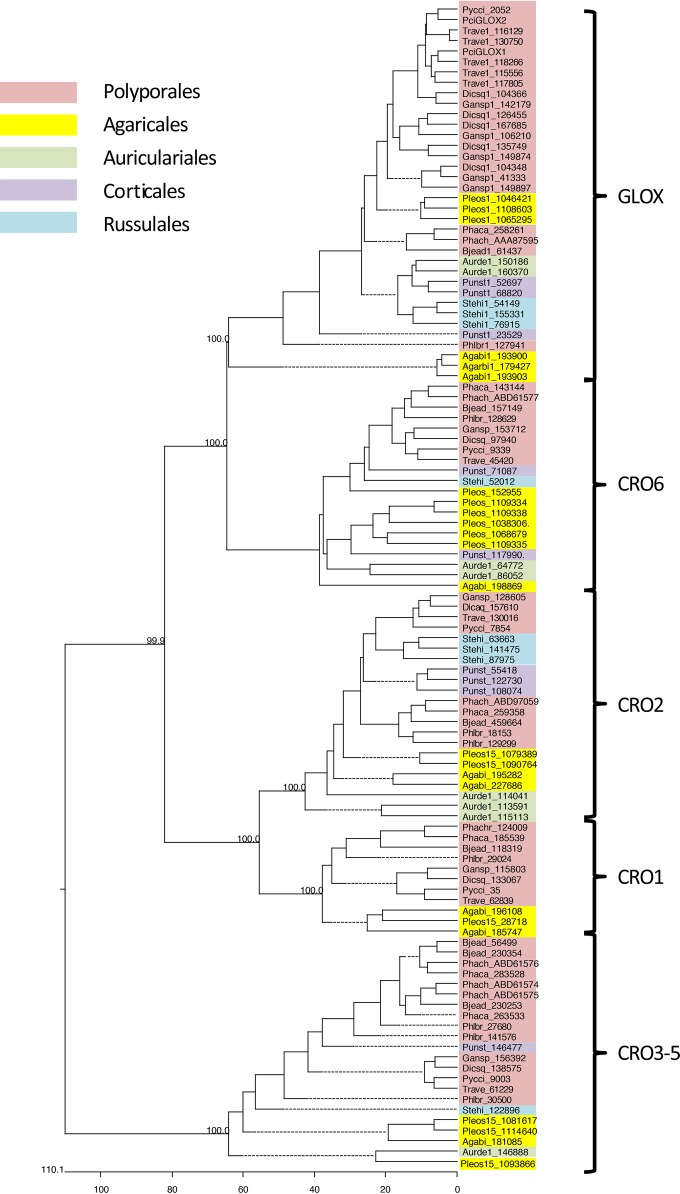
Phylogenetic tree showing relationships among predicted CRO proteins derived from representative white rot fungi, including P. cinnabarinus and P. chrysosporium. ClustalW alignments and tree construction followed the manual deletion of secretion signals and, in the case of CRO3 to CRO5, the removal of repetitive N-terminal WSC domains. On the basis of 1,000 trials, bootstrap values are shown at key nodes. Substitutions were calculated by use of the Kimura distance formula. Taxonomic orders are highlighted by colored shading. Trave, Trametes versicolor; Dicsq, Dichomitus squalens; Gansp, a Ganoderma sp.; Phaca, Phanerochaete carnosa; Bjead, Bjerkandera adusta; Phlbr, Phlebia brevispora; Pci and Pycci, Pycnoporus cinnabarinus; Pleos, Pleurotus ostreatus; Agabi, Agaricus bisporus; Aurde, Auricularia delicata; Punst, Punctularia strigozonata; Stehi, Stereum hirsutum.

**FIG 2:**
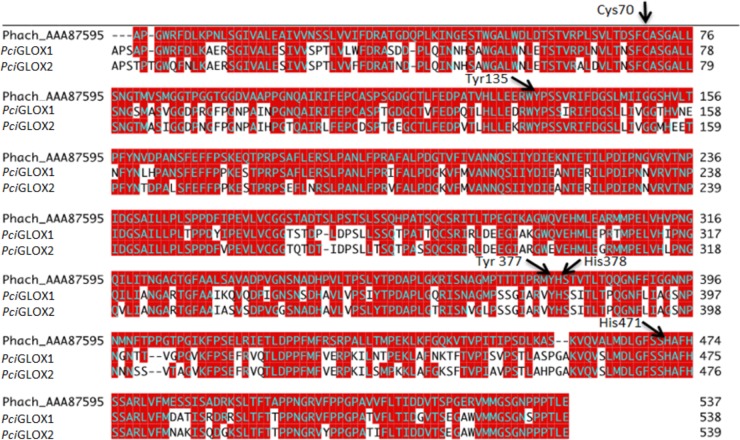
ClustalW alignment of highly expressed Pycnoporus cinnabarinus (*Pci*) GLOX-like proteins with P. chrysosporium (Phach) GLOX. Identical residues are highlighted in red. Arrows, residues essential for catalysis.

*Pci*GLOX sequences (*Pci*GLOX1, *Pci*GLOX2, and *Pci*GLOX3, referred to as Pycci_2052 in Fig. 1) were confidently embedded within the GLOX clade among other Polyporales (Fig. 1). The genomic sequences of both *Pci*GLOX1 and *Pci*GLOX2 contained 3 introns with strong position homology and signal peptide sequences of 18 and 20 amino acids, respectively. The cDNA sequences of *Pci*GLOX1 and *Pci*GLOX2 consisted of open reading frames of 1,671 and 1,680 bp encoding 556- and 559-amino-acid proteins, respectively. The identity between the two *Pci*GLOX proteins was 85%, and the amino acids at the active site were found to be highly conserved (Fig. 2).

### Protein production and purification.

The codon-optimized gene sequences containing an upstream GLA prepro sequence from A. niger and a downstream N-terminal 6×His tag were cloned in the pAN52.4 vector. The plasmids were used for the transformation of A. niger protoplasts, and positive clones were selected and used for the large-scale production of *Pci*GLOX1 and *Pci*GLOX2. Protein production was followed at days 7, 9, and 11 by measuring activity against methylglyoxal in the culture supernatant. An increase in the total activity measured from 9,093 nkat to 43,293 nkat was observed during *Pci*GLOX1 production; however, *Pci*GLOX2 activity was not detectable in the culture medium before ultrafiltration. The recombinant *Pci*GLOX1 and *Pci*GLOX2 proteins were purified 20.9- and 607.8-fold using IMAC, yielding 59 mg and 5 mg, respectively, from 600 ml of culture (Table 1). The purified protein was green and turned into light purple after dialysis against sodium phosphate (pH 7) buffer. The purified proteins were shown to be homogeneous using SDS-PAGE (see Fig. S1a in the supplemental material). The molecular mass was estimated to be 70 kDa for both enzymes (see Fig. S1a in the supplemental material). The N-terminal amino acid sequences obtained were APSA and APST for *Pci*GLOX1 and *Pci*GLOX2, respectively, which correspond to the predicted sequences. These results show that the recombinant proteins were properly processed from the N terminus.

**TABLE 1:** Purification of *Pci*GLOX1 and *Pci*GLOX2

Purification step	Vol (ml)		Total activity (nkat)		Amt of protein (mg)		Sp act (nkat · mg^−1^)		Activity yield (%)		Purification factor (fold)	
	*Pci*GLOX1	*Pci*GLOX2	*Pci*GLOX1	*Pci*GLOX2	*Pci*GLOX1	*Pci*GLOX2	*Pci*GLOX1	*Pci*GLOX2	*Pci*GLOX1	*Pci*GLOX2	*Pci*GLOX1	*Pci*GLOX2
Culture medium	600	600	43,293	ND^*a*^	150	114	288	ND	100	ND	1	ND
Ultrafiltration	100	100	50,850	1,600	134	167	379	9	118	100	1.3	1
IMAC purification	7	10	355,159	27,350	59	5	6,019	5,470	822	1,709	20.9	607.8

a ND, not detected.

The obtained molecular mass of 70 kDa was larger than the predicted molecular mass for both proteins (58 kDa calculated using ProtParam-ExPaSy), suggesting the presence of glycosylation. The NetNGlyc (version 1.0) server (accessible via http://www.cbs.dtu.dk/services/NetNGlyc) predicted 5 and 6 N-glycosylation sites for *Pci*GLOX1 and *Pci*GLOX2, respectively, supporting the results observed with SDS-PAGE. The presence of N-glycosylation sites was confirmed by treating both enzymes with PNGase F, and the resulting deglycosylated proteins were about 60 kDa in mass, corresponding to the theoretical mass of the *Pci*GLOX proteins (see Fig. S1b in the supplemental material). On the basis of the findings on SDS-polyacrylamide gels, the glycosylation percentage was about 14% for both enzymes. The two *Pci*GLOX proteins had a pI of 6 (data not shown). This result was in agreement with the pI predicted from the cDNA sequences (5.8 for *Pci*GLOX1 and 5.5 *Pci*GLOX2).

### Effect of pH and temperature.

**FIG 3:**
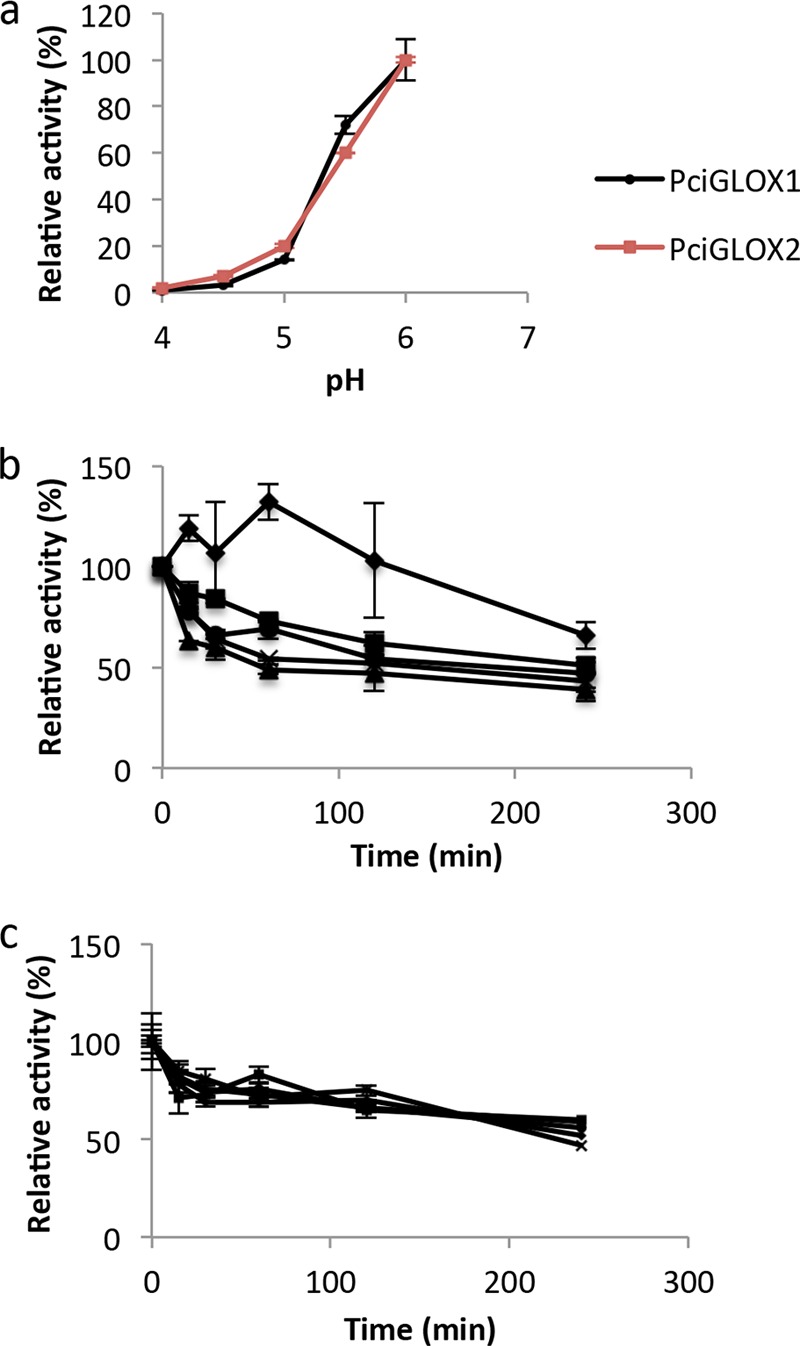
(a) Effect of pH on activity of purified *Pci*GLOX1 and *Pci*GLOX2. Relative activity represents a percentage of the activity at the maximum pH. (b, c) Effect of pH 4 (◆), pH 4.5 (■), pH 5(▲), pH 5.5 (×), and pH 6 (•) on the enzymatic stability of *Pci*GLOX1 (b) and PciGLOX2 (c) after different incubation periods. The relative activity after incubation for each pH condition was calculated as a percentage of the activity at the same pH without incubation.

The two *Pci*GLOX enzymes showed narrow optimum activity at pH 6 (Fig. 3a), with only 20% relative activity at pH 5. The pH stability was determined by incubating the enzymes in buffer at a pH of between 4 and 6 for 15, 30, 60, 120, and 240 min. The two *Pci*GLOX enzymes were found to lose activity gradually with time, with only 40% and 60% activity remaining after 4 h of incubation at 30°C (Fig. 3b and c). The extent of activity loss over time was similar for all tested pH conditions.

**FIG 4:**
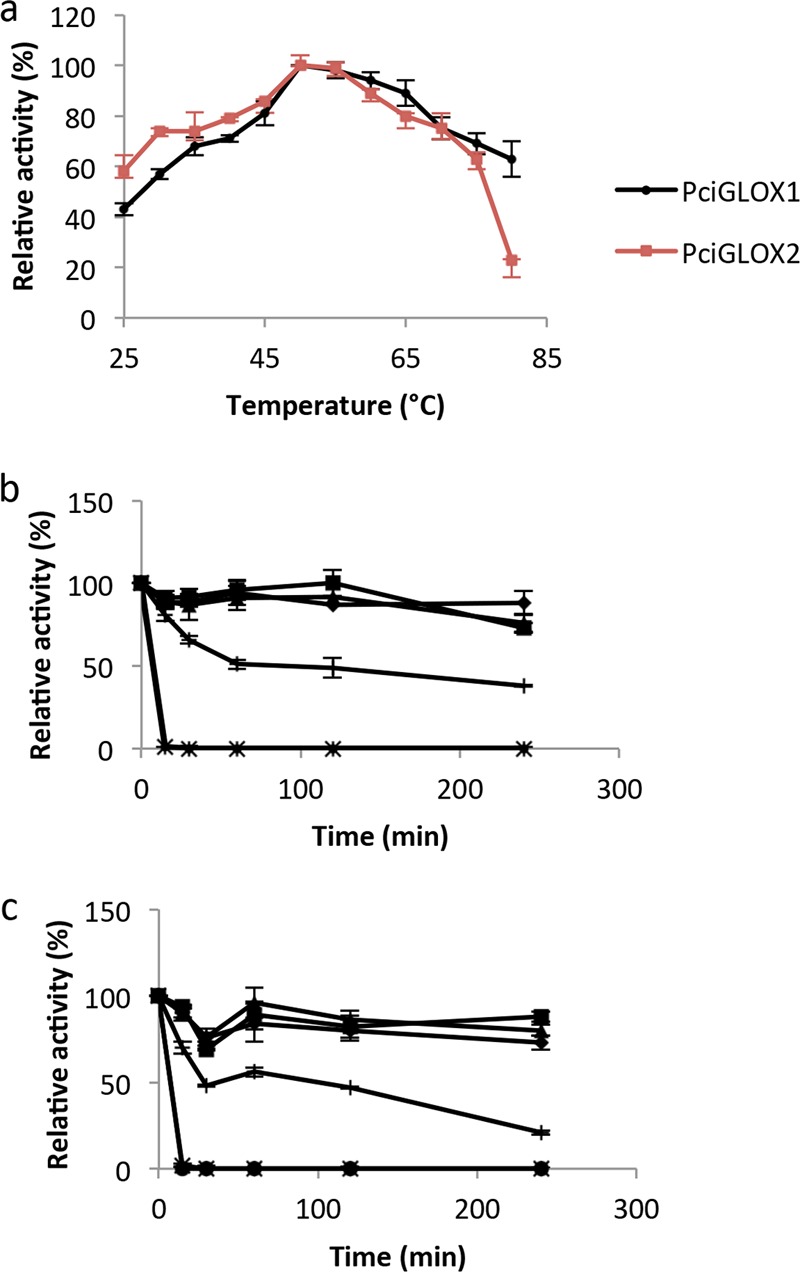
(a) Effect of temperature on enzymatic activity of *Pci*GLOX1 and *Pci*GLOX2. Values were calculated as a percentage of the activity at the maximum temperature. (b, c) The protein stability of *Pci*GLOX1 (b) and *Pci*GLOX2 (c) at 30°C (◆), 40°C (■), 50°C (▲), 60°C (+), 70°C (∗), and 80°C (•) after incubation for different periods of time is represented as a percentage of the activity at 30°C without incubation.

The optimum temperature was found to be 50°C for both enzymes, which had similar temperature profiles (Fig. 4a). Both enzymes retained 60% activity at 75°C. Thermal stability (at 30 to 80°C) was also determined by incubating the enzymes for 15, 30, 60, 120, and 240 min at these temperatures. The two enzymes behaved in a similar way at the tested temperatures (Fig. 4b and c). Only about 20% of the activity was lost after 4 h of incubation at 50°C. The proteins retained 50% of their activity after 2 h at 60°C and completely lost activity after a 15-min incubation at 70° and 80°C.

### Substrate specificity and kinetics.

The two *Pci*GLOX enzymes showed broad substrate specificity (Table 2). Specifically, *Pci*GLOX2 was active on a wider number of substrates than *Pci*GLOX1. *Pci*GLOX2 was more active than *Pci*GLOX1 on dl-glyceraldehyde, dihydroxyacetone, glycerol, and formaldehyde and was the only one active on 3-phenylpropionaldehyde. The highest activity was found on glyoxylic acid (192% for *Pci*GLOX1 and 132% for *Pci*GLOX2 relative to their activities on methylglyoxal). Neither of the enzymes was able to oxidize sugars or methanol. The kinetic parameters of both enzymes on selected substrates are presented in Table 3. The highest catalytic efficiency was found for glyoxylic acid (2,136.363 s^−1^ mM^−1^ for *Pci*GLOX1 and 17 s^−1^ mM^−1^ for *Pci*GLOX2), followed by methylglyoxal, glyoxal, and glycerol. The catalytic efficiency of *Pci*GLOX1 for methylglyoxal, glyoxal, and glyoxylic acid was significantly higher than that of *Pci*GLOX2.

**TABLE 2:** Substrate specificity of *Pci*GLOX1 and *Pci*GLOX2^*a*^

Substrate	Relative activity (%)	
	*Pci*GLOX1	*Pci*GLOX2
Methylglyoxal	100	100
Glyoxal	40	56
Glyoxylic acid	192	132
3-Phenylpropionaldehyde	ND^*b*^	46
Formaldehyde	6	65
Veratraldehyde	ND	ND
4-Hydroxybenzaldehyde	ND	ND
2,4-Dimethoxybenzaldehyde	ND	ND
dl-Glyceraldehyde	1	22
Dihydroxyacetone	13	54
Phenyl glyoxylic acid	ND	ND
Formic acid	ND	ND
d-Glucose	ND	ND
d-Galactose	ND	ND
d-Xylose	ND	ND
Glycerol	ND	24
Methanol	ND	ND

a The standard activity test was performed with 10 mM substrate, and the values were calculated relative to those for methylglyoxal.

b ND, not detected under these conditions.

**TABLE 3:** Kinetic parameters of *Pci*GLOX1 and *Pci*GLOX2 with different substrates^*a*^

Substrate	*Pci*GLOX1				*Pci*GLOX2			
	*V*_max_ (nkat mg^−1^)	*K_m_* (mM)	*k*_cat_ (s^−1^)	*k*_cat_/*K_m_* (s^−1^ mM^−1^)	*V*_max_ (nkat mg^−1^)	*K_m_* (mM)	*k*_cat_ (s^−1^)	*k*_cat_/*K_m_* (s^−1^ mM^−1^)
Methylglyoxal	1,289	1.3	76	58.461	245	0. 2	1.4	7
Glyoxal	1,422	13.1	83	6.335	250	2.2	1.4	0.636
Glyoxylic acid	3,193	0.088	188	2,136.363	298	0.1	1.7	17
Glycerol	523	660.5	33	0.049	107	9.4	0.6	0.063

a The standard test was used; however, the concentration of substrate was varied.

The conversion of methylglyoxal, glyoxal, and glyoxylic acid by both *Pci*GLOX enzymes to their corresponding carboxylic acids was analyzed by HPLC. When glyoxylic acid was used as the substrate, a decrease in the peak representing glyoxylic acid was observed, and a new peak corresponding to oxalic acid appeared at 8.97 min (see Fig. S2a in the supplemental material). In the case of glyoxal as the substrate, a peak corresponding to oxalic acid was seen for the reaction by the two enzymes, while a second peak corresponding to glyoxylic acid appeared only in the *Pci*GLOX2 reaction (see Fig. S2b in the supplemental material). In the reactions with methylglyoxal, a peak corresponding to pyruvic acid was present (see Fig. S2c in the supplemental material).

## DISCUSSION

Two glyoxal oxidases from P. cinnabarinus were heterologously produced in A. niger and biochemically characterized. The genes encoding these proteins, along with the *glox3* gene, were found on the same scaffold and positioned in the same transcriptional direction, thus forming a cluster (4). In P. chrysosporium, three CRO-encoding genes related to but structurally distinct from the GLOX gene were grouped on the same scaffold along with eight class II peroxidases (3). However, in P. cinnabarinus, the *glox* and peroxidase clusters were located on different scaffolds (4).

By comparing the sequences of the two *Pci*GLOX proteins described in this paper and the previously characterized GLOX from P. chrysosporium (GenBank accession no. AAA87595, referred to as *Ph*GLOX in this paper) (20), a 74% similarity was found (Fig. 1). The alignment further showed that the amino acid residues responsible for ligating the copper ion active site and characteristic of CROs (Cys73, Tyr138, His380, Tyr379, and His473) (21) were highly conserved in the *Pci*GLOX and *Ph*GLOX proteins. These residues are also conserved in galactose oxidases (22), suggesting that GLOX and galactose oxidases should follow a similar mechanism of action for the oxidation of their substrates. This might be explained by the distinctive function of the radical copper complex of these enzymes acting as a two-electron redox-active site, in contrast to other free radical enzymes that show single-electron reactivity, such as ribonucleotide reductase (8).

The production of GLOX proteins in Aspergillus species was previously performed by Kersten and Cullen (23), and the yield of proteins was between 10 and 20 mg per liter of culture, similar to the yield obtained for *Pci*GLOX2 in this study. However, a five times larger amount of *Pci*GLOX1 was obtained. Before buffer exchange the purified proteins were green. This color was previously assigned to the oxidized active form of the protein (24). The purple form of the enzyme obtained after dialysis against 50 mM sodium phosphate (pH 7) was also reported previously when the enzyme was dialyzed into potassium phosphate at the same pH and down to pH 4.53 (24). The two purified glycosylated proteins were similar in size to *Ph*GLOX (68 kDa), which was also found to be N-glycosylated, with glycosylation accounting for about 16% of the total protein mass (12, 23). Contrary to the optimum temperature for *Ph*GLOX, which is 30°C (25), the optimum temperature for both *Pci*GLOX described here was 50°C, and activity was still detected at 75 to 80°C. The two *Pci*GLOX enzymes were found to be similarly affected by temperature and were relatively stable at 50°C after 4 h of incubation. The activities of both enzymes were found to be highly dependent on the buffer used, since the use of different buffers with the same pH was found to alter the activities of the enzymes. For this reason, the pH profile test was limited to the pH range of sodium 2.2-dimethylsuccinate (pH 4 to 6), the buffer in which both enzymes were found to be the most active. In addition, over this pH range, there was no effect on HRP activity (18). The optimum pH obtained under the tested conditions for both *Pci*GLOX enzymes was 6, which is in agreement with that previously reported for *Ph*GLOX (26, 27). However, our results differ from those obtained by Son et al. (25), who showed that the optimum pH for the recombinant *Ph*GLOX was 5. In our study, it was noticed that the enzymes were very sensitive to minor pH changes and they were less active at pH 5, which is a change of only 1 pH unit from the optimal pH. However, the results showed that the enzymes retained about 50% of their activity after incubation for 4 h under all tested pH conditions.

Similar to *Ph*GLOX (12), a 1.4-fold increase in activity was observed when *Pci*GLOX1 was incubated with 1 mM CuSO_4_ for 30 min at 25°C. However, no similar effect on the activity was detected for *Pci*GLOX2. This might be explained by the fact that the large amount of *Pci*GLOX1 produced in the culture medium requires more copper ions to ensure complete ion activation. The presence of copper(II) ions (e.g., in the form of CuSO_4_) has previously been found to be required for the activation of galactose oxidase (28).

The two purified GLOX proteins were active only in the presence of both HRP and ABTS in the reaction mixture, as HPLC analysis demonstrated (data not shown). *Pci*GLOX appears to be activated when the enzyme is oxidized by one electron or the copper is reduced by one electron, as has been previously proposed for *Ph*GLOX (8). This activation mechanism is thought to be directed by lignin peroxidase and veratryl alcohol (12). Furthermore, the activation by peroxidase (lignin peroxidase or HRP) was reported to be possible only when a high-redox-potential peroxidase substrate was used in the reaction (29). The activation of GLOX by peroxidase might be a mechanism used by fungi to regulate peroxide generation and thus control peroxidase action *in vivo* (24, 29). GLOX has also been found to be activated by high-redox-potential inorganic oxidants, including manganese chelates (Mn^3+^ EDTA) (24), possibly relating this enzyme to manganese peroxidase, which is responsible for the generation of Mn^3+^. HPLC analysis also showed that the substrates were not completely converted even when the reaction time was increased, suggesting that the enzyme is inactivated at some point during the reaction. This inactivation has been attributed in part to the inhibitory effect of peroxide accumulation on GLOX (29). Further investigations by Roncal et al. (27) showed that the inactivation was triggered by components that are not initially present in the reaction medium or that are present in a different state at first. These components could be products of the reaction or other generated compounds directly inhibiting the enzyme or interfering with its mechanism of action under the coupled reaction conditions (27).

The two *Pci*GLOX enzymes had broad substrate specificity. The enzymes were unable to oxidize sugars and methanol, showing that they are not sugar or methanol oxidases. Substrates included methylglyoxal, glyoxal, glyoxylic acid, glyceraldehyde, dihydroxyacetone, and glycerol, which are compounds derived from carbohydrate metabolism. The nature of the substrates could explain the production of *Ph*GLOX when the fungus was grown on glucose and xylose as the main carbohydrates in the culture medium (23). Similarly, *Pci*GLOX1 and *Pci*GLOX2 were detected in the secretome of the fungus when it was grown on maltose as the carbon source (4). Another potential source of GLOX substrates is through lignin degradation itself by the consecutive oxidation of glycoaldehyde to glyoxal and then glyoxylic acid (5). Glyoxal can also be formed through lipid peroxidation of linoleic acid by manganese peroxidase (30), again linking GLOX with the ligninolytic peroxidases in fungi. A major difference between the two *Pci*GLOX enzymes and *Ph*GLOX is that the best substrate for the former was glyoxylic acid instead of methylglyoxal. Both GLOX enzymes were active in the majority of the reported substrates of *Ph*GLOX (26, 27); however, *Pci*GLOX2 was also found to act on 3-phenylpropionaldehyde. Activity on glycerol is interesting, since it is a sugar alcohol lacking the formyl group present in the other substrates. Glycerol is structurally similar to dihydroxyacetone, a molecule found to be a substrate for *Ph*GLOX (26) and *Pci*GLOX. It has also been proposed that the gem diol form obtained after hydration of aldehydes to acetals is the actual substrate recognized by GLOX (24). Primary alcohols are structural analogues of gem diols, which derive from aldehydes, and this might explain the activity of GLOX on glycerol (27). In a similar way, carbonyl-containing substrates are suggested to be hydrated by solvent coordinated to the copper in the active site, and this form of the substrates is recognized by the enzyme (24).

In some cases, the *Pci*GLOX enzymes were able to also act on the primary products of their reactions (see Fig. S2 and S3 in the supplemental material). As shown by HPLC, while the only product of *Pci*GLOX1 on glyoxal was oxalic acid, which is the final product of the reaction, in the case of *Pci*GLOX2, another peak corresponding to glyoxylic acid, the intermediate compound of this reaction, was also detected (see Fig. S2b in the supplemental material). The absence of the first product in the reaction of *Pci*GLOX1 can be explained by the higher catalytic efficiency (more than 100 times) of this enzyme compared to that of *Pci*GLOX2 in oxidizing this compound (Table 3). These findings suggest that the *Pci*GLOX enzymes are implicated in sequential reactions or their substrate preference evolves during fungal growth. This characteristic could be biotechnologically important, since it is possible to perform multiple oxidation steps using one enzyme. Recently, Yin and coworkers (31) showed that two fungal alcohol oxidases from Colletotrichum graminicola and Colletotrichum gloeosporioides belonging to the AA5_2 family were incapable of oxidizing sugars but were efficient in the oxidation of a diverse number of aliphatic alcohols, including glycerol. Considering the broad specificity of *Pci*GLOX2, oxidases belonging to the AA5 family could be rather promiscuous regarding their substrate specificity.

As a hydrogen peroxide-generating enzyme, GLOX is thought to be part of the lignin-degrading enzyme machinery by providing H_2_O_2_ for peroxidase activity. Recently, it was shown that, when P. chrysosporium was grown on solid spruce wood, a significant upregulation of manganese and lignin peroxidases and auxiliary enzymes, including GLOX, was observed during extracellular oxidation, which was detected by the presence of oxidant-sensing beads bearing a fluorometric dye (32). However, secretomic analysis of P. cinnabarinus cultures on different substrates does not clearly establish the relation between these enzymes since peroxidases were detected only in solid-state fermentation, whereas the *Pci*GLOX enzymes were present in liquid and solid cultures (4). On the other hand, the activation by peroxidases reported previously and also in this work could support the synergistic activity between GLOX and peroxidases. The amino acids involved in copper binding were highly conserved in the *Pci*GLOX enzymes, as well as in *Ph*GLOX, which might explain the similar characteristics between these enzymes. However, the *Pci*GLOX enzymes were more active on glyoxylic acid, contrary to the findings for *Ph*GLOX, whose best substrate was methylglyoxal. In addition, a difference in catalytic efficiency and substrate preference was observed between the *Pci*GLOX1 enzymes and *Pci*GLOX2, which either acted alone or more efficiently on some of the substrates. In light of these differences, the multiplicity of GLOX genes in P. cinnabarinus may reflect an adaptation to shifting substrate composition and availability during decay of complex woody cell walls. A better understanding of the 3-dimensional structure and the mechanism of action of these enzymes is needed to explain the results obtained in this study.

## Supplementary Material

Supplemental material
