# Supplementary material for: Heterologous Production and Characterization of Two Glyoxal Oxidases from Pycnoporus cinnabarinus
Source: Appl Environ Microbiol. 2016 Jul 29;82(16):4867–75. doi: 10.1128/AEM.00304-16 (PMC4968546; doi:10.1128/AEM.00304-16)
Supplement: Supplemental material [file supp_82_16_4867__index.html]

Supplemental material 

# Heterologous Production and Characterization of Two Glyoxal Oxidases from Pycnoporus cinnabarinus

## Supplemental material

- Supplemental file 1 -

  SDS-PAGE analysis of purified proteins and of N-deglycosylation of *Pci*GLOX1 and *Pci*GLOX2 with PNGase F (Fig. S1); HPLC analysis of *Pci*GLOX1 and *Pci*GLOX2 oxidation of glyoxal, glyoxylic acid, and methylglyoxal (Fig. S2); proposed reactions catalyzed by glyoxal oxidase (Fig. S3).

  PDF, 449K
