# Supplementary material for: Heterologous Production and Characterization of Two Glyoxal Oxidases from Pycnoporus cinnabarinus
Source: Appl Environ Microbiol. 2016 Jul 29;82(16):4867–75. doi: 10.1128/AEM.00304-16 (PMC4968546; doi:10.1128/AEM.00304-16)
Supplement: Supplemental material [file AEM.00304-16_zam999117337so1.pdf]

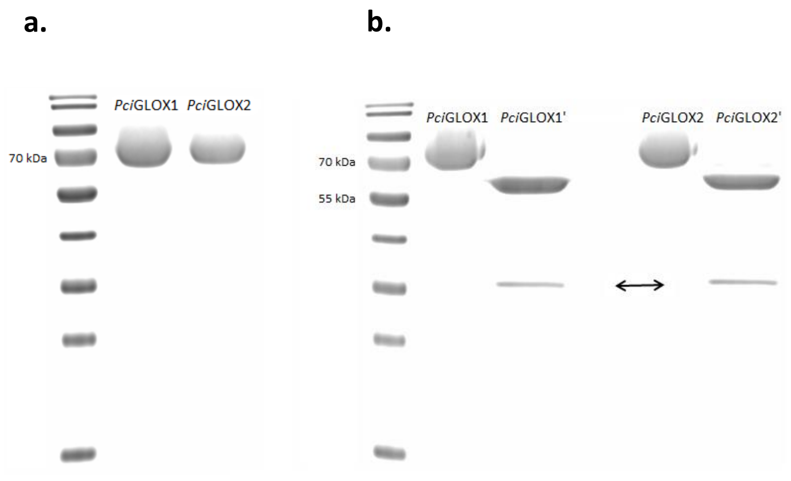

Fig. S1. (**a**) SDS-analysis of purified proteins. (**b**) N-deglycosylation of *Pci*GLOX1 and *Pci*GLOX2 with PNGase F which has a molecular weight of 36 kDa and is also visible on the gel (arrow). Lane M, standard marker.

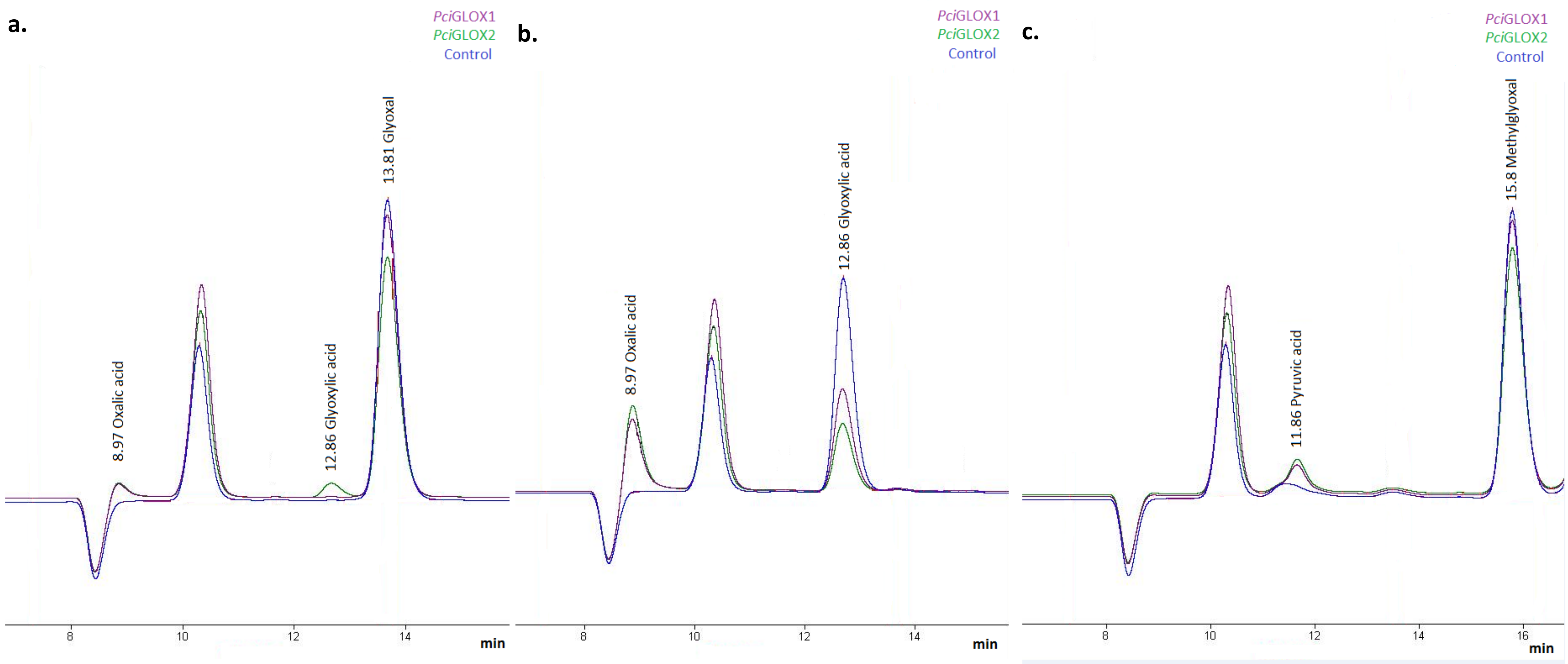

Fig. S2. HPLC analysis of *Pci*GLOX1 and *Pci*GLOX2 oxidation of (**a**) glyoxal, (**b**) glyoxylic acid and (**c**) methylglyoxal. The reactions were carried out as described in the method section.

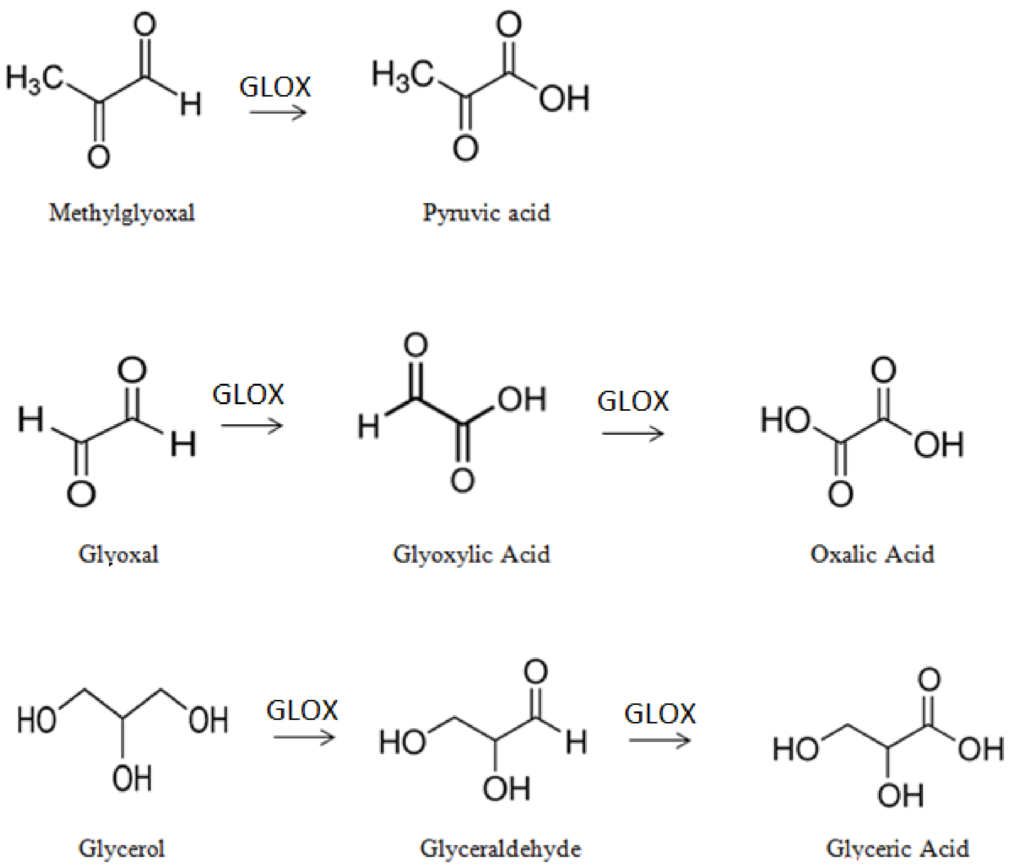

Fig. S3. Proposed reactions catalyzed by glyoxal oxidase.
